# Innovative Strategies for Team Treatment Planning and Collaborative Goal-Setting in Home-Based Primary Care

**DOI:** 10.1093/geroni/igaf122.052

**Published:** 2025-12-31

**Authors:** Tara Afonso, Michelle Mlinac, Katie Mendoza

## Abstract

Collaborative treatment planning with older adults and caregivers in home-based primary care (HBPC) can be challenging for the interprofessional teams who serve them. This symposium describes a multi-faceted stepped quality improvement approach to changing the process of interdiscplinary treatment planning (IDTP) within an HBPC program. This program spans four individual teamlets and has a patient census of 580. Members of the interdisciplinary team include nurses, social workers, physicians, nurse practitioners, physicians assistants, pharmacists, dieticians, rehab therapists, medical support assistants, and psychologists. The first presentation provides an overview of the strategic planning course for the team which began with a revamp of the structure and workflow of the IDTP meeting. Planful documentation improvements include the creation of an overall ‘mission’ for each patient to help guide the course of longitudinal care, as well as enhancing documentation of the 4M’s to underscore holistic geriatric care delivery. The second presentation describes the approach to foster the spirit of motivational interviewing (MI) within the team. In order to facilitate learning and promote buy-in, a gamification strategy was employed, and results suggest both immediate and sustained learning gains. The third presentation provides a review of how the team elicited patient SMART goals, aligned them with team treatment goals, and worked towards achievement of those goals. The strategic use of gamification was again enlisted to foster a friendly competition among the four teamlets to adopt new skills in a safe learning environment. Overall this multi-year QI process has driven person-centered care improvements for patients with multicomplexity.

